# Feasibility of Serial 6-min Walk Tests in Patients with Acute Heart Failure

**DOI:** 10.3390/jcm6090084

**Published:** 2017-09-11

**Authors:** Sean P. Collins, Michael Thorn, Richard M. Nowak, Phillip D. Levy, Gregory J. Fermann, Brian C. Hiestand, Tillman Douglas Cowart, Robert P. Venuti, William R. Hiatt, ShiYin Foo, Peter S. Pang

**Affiliations:** 1 Department of Emergency Medicine, Vanderbilt University Medical Center and Tennessee Valley Healthcare System, Nashville, TN 37232, USA; 2 Statistical Resources, Inc., Chapel Hill, NC 27514, USA; mthorn@statisticalresources.com; 3 Department of Emergency Medicine, Henry Ford Health System, Detroit, MI 48126, USA; rnowak1@hfhs.org; 4 Department of Emergency Medicine and Integrative Biosciences Center, Wayne State University, Detroit, MI 48201, USA; plevy@med.wayne.edu; 5 Department of Emergency Medicine, University of Cincinnati, Cincinnati, OH 45267, USA; fermangj@ucmail.uc.edu; 6 Wake Forest School of Medicine, Winston Salem, NC 27109, USA; bhiestan@wakehealth.edu; 7 Therapeutic Development Consultants LLC; dcowart@theradevel.com; 8 Previously of Cardioxyl Pharmaceuticals, Chapel Hill, NC 27514, USA; rvenuti@nc.rr.com; 9 Division of Cardiology, University of Colorado School of Medicine and CPC Clinical Research, Aurora, CO 80045, USA; will.hiatt@cpcmed.org; 10 Orchard Biomedical Consulting LLC, Brookline, MA 02446, USA; shiyinfoo@gmail.com; 11 Department of Emergency Medicine & Indianapolis EMS, Indiana University School of Medicine, Indianapolis, IN 46202, USA; ppang@iu.edu

**Keywords:** 6 min walk test, acute heart failure, emergency department

## Abstract

Background: Functional status assessment is common in many cardiovascular diseases but it has undergone limited study in the setting of acute heart failure (AHF). Accordingly, we performed a pilot study of the feasibility of the six-minute walk test (6MWT) at the emergency department (ED) presentation and through the hospitalization in patients with AHF. Methods and Results: From November 2014 to February 2015, we conducted a multicenter, observational study of ED patients, aged 18–85 years, whose primary ED admission diagnosis was AHF. Other criteria for enrollment included a left ventricular ejection fraction ≤40%, systolic blood pressure between 90 and 170 mmHg, and verbal confirmation that the patient was able to walk >30 m at the baseline, prior to ED presentation. Study teams were uniformly trained to administer a 6MWT. Patients underwent a baseline 6MWT within 24 h of ED presentation (Day 1) and follow-up 6MWTs at 24 (Day 2), 48 (Day 3), and 120 h (Day 5). A total of 46 patients (65.2% male, 73.9% African American) had a day one mean walk distance of 137.3 ± 78 m, day 2 of 170.9 ± 100 m, and day 3 of 180.8 ± 98 m. The 6MWT demonstrated good reproducibility, as the distance walked on the first 6MWT on Day 3 was similar to the distance on the repeated 6MWT the same day. Conclusions: Our pilot study demonstrates the feasibility of the 6MWT as a functional status endpoint in AHF patients. A larger study in a more demographically diverse cohort of patients is necessary to confirm its utility and association with 30-day heart failure (HF) events.

## 1. Background

Quality of life is critical to patients’ health and well-being. While reducing readmissions and mortality remain important endpoints, maintaining or improving the quality of life may be more important to patients. For heart failure (HF) patients, some would trade life years for a better life quality [1]. One key component of quality of life for HF patients is functional ability, such as maintaining the activities of daily living and self-care.

Functional status assessment is commonly used in chronic HF studies and other cardiovascular diseases [2]. While subjective symptom assessments are common in acute heart failure (AHF), however, clinical trials and formal functional status assessment are not [3,4]. Ideally, AHF patients return to their baseline functional capacity prior to discharge. However, this is seldom rigorously evaluated. In chronic HF, the 6 min walk test (6MWT) is a validated measure of functional capacity, assessing the distance a patient can walk in 6 min, performed at the patient’s intrinsic walking speed, including all patient-determined stops. Changes in 6MWT distance as a result of therapeutic interventions predict rehospitalizations and mortality [5,6,7]. However, the feasibility of performing baseline and serial 6MWT in AHF, to assess the potential response to therapy has not been well studied [8]. We performed a pilot study to determine the feasibility of performing the 6MWT in AHF patients in the emergency department (ED) and continuing through hospitalization.

## 2. Methods

**Study Design and Setting:** From November 2014 to February 2015, we conducted a multicenter observational study of ED patients, aged 18–85 years, whose primary ED admission diagnosis was AHF. Patients were enrolled at six academic, urban hospitals in the United States. The study and all associated procedures were approved by local and/or central IRBs/ethics committees per each participating institution, and all patients provided written informed consent. 

**Participants:** AHF was established at each institution by the ED provider using history and physical exam findings, congestion on chest radiograph, and elevated levels of natriuretic peptides. This diagnosis was confirmed with the treating physician by the study team prior to approaching the patient. All patients were enrolled within 24 h of ED presentation, though the investigators were encouraged to approach the patients as soon as AHF was confirmed by the provider. The remaining inclusion criteria included a left ventricular ejection fraction (LVEF) ≤40% (as assessed by echocardiography, MRI, MUGA, or right heart catheterization within the 6 months prior to the ED presentation), systolic blood pressure (SBP) between 90 and 170 mmHg, and verbal confirmation that the patient was able to walk at least 30 m prior to developing AHF symptoms leading to the index presentation. Patients were excluded who had other significant non-cardiac limitations to their ambulatory capacity, who were considered likely to undergo an invasive procedure within three days of study enrollment or who had eGFR <30 mL/min/1.73 m^2^. 

**Study Procedures:** Research teams at all six institutions were uniformly trained by using standardized methods by study personnel from the Colorado Prevention Center Clinical Research, a group with extensive prior 6MWT trial experience [9]. Study teams reviewed vital signs and the most recent laboratories prior to commencing the 6MWT to identify clinically significant changes that might preclude performance of the 6MWT, such as the orthostatic or new symptomatic systolic blood pressure less than 90 mmHg. Per eligibility criteria, no patient with a SBP <90 mmHg was allowed to participate. Furthermore, the investigators were specifically told to exclude patients with any other significant lab abnormalities that would increase the risk associated with the 6MWT. Oxygen therapy or walking aids required at baseline were continued in a similar fashion in all subsequent tests for any given patient. Patients enrolled in the study underwent a baseline (Day 1) 6MWT as early as possible while in the ED; serial 6MWTs were performed at 24 (Day 2), 48 (Day 3), and 120 h (Day 5; or at discharge, if sooner) during the patient’s hospital stay. At the 48-h time point, two sequential 6MWTs were performed, separated by a 30-min rest between the tests. Distance to the first stop was defined as the total distance (meters) walked by the patient before indicating they needed to rest. Prior to conducting the 6MWT, patient safety was assessed by evaluating the serial vital signs, including assessments immediately preceding the performance of the 6MWT. Routine laboratories, BNP (B-type natriuretic peptide) or NTproBNP, and ECGs were monitored after the 6MWT as well. Data on rehospitalizations and mortality were collected up to Day 35 after enrollment, or 30 days after discharge from the hospital if the index length of stay was less than 5 days. 

**Statistical Analysis:** The sample size for this pilot observational study was based on prior observations. Approximately 50–70 patients per arm [10] have previously been required to demonstrate a statistically significant change in the 6MWT due to an intervention in other ill patient populations. Patients who declined any attempt to perform the 6MWT (outside of the baseline) based on personal preference were not included in the analysis for that specific time point. A distance of 0 m for the 6MWT at any given time point was imputed to patients who were willing but unable to perform the 6MWT at that time point due to deteriorations in clinical status. Means and other descriptive statistics were calculated, and a correlation between baseline 6MWT and subsequent 6MWT performance, hospital length of stay (LOS), BNP levels, and 30-day events were analyzed using SAS system software (GitHub, Cary, NC, USA), version 9.4 on a Windows 10 platform. Cox proportional hazards modeling was used to evaluate the association between 6MWT performance and hospital readmission within 30 days. 

## 3. Results

### 3.1. Participant Characteristics 

**Figure 1:**
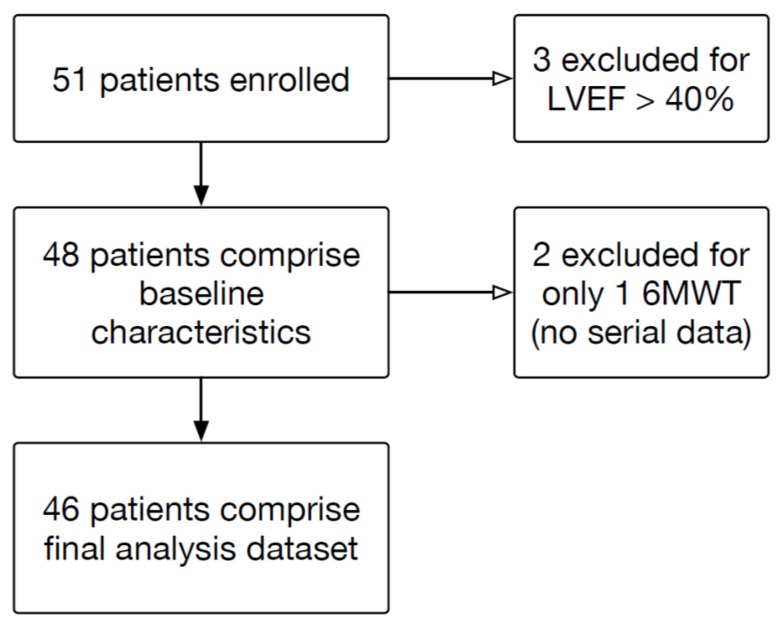
Enrollment and patient flow through the study.

Figure 1 demonstrates the study allocation of the 51 patients enrolled. There were 15 (29.4%) patients enrolled at Center 1, 25 (49.0%) at Center 2, 3 (5.9%) at Center 3, one (2.0%) at Center 4, 6 (11.8%) at Center 5, and one (2.0%) at Center 6. Of the 48 patients with LVEF ≤40%, their mean age was 55.8 ± 12.9 years, 65.2% were male, and 73.9% of patients were African-American (Table 1). Mean LVEF was 24.1 ± 9.2% and mean ED systolic blood pressure was 126.9 ± 19.7 mmHg (range 90–149 mmHg). The mean time from presentation to performance of the first, baseline 6MWT was 10.8 ± 5.6 h (range 2.1 to 21.8 h). Forty-six patients had serial walk tests available and were included in the final analysis cohort. The majority (85%) of patients received intravenous diuretics during their ED stay, while 15% received topical or sublingual nitrates. 

### 3.2. Safety and Feasibility of 6MWT 

Of the 46 total patients, 44 (95.7%) attempted a baseline 6MWT (Day 1). Of these, 21 (45.7%) required at least one rest stop prior to the end of the 6-min walk period. Fewer patients attempted a 6MWT on Day 2 (*n* = 38) and Day 3 (*n* = 28), largely due to early hospital discharge, while a minority were unable to due to worsening clinical status. Importantly, only 4 patients refused to do a total of 5 of the 154 individual 6MWTs in this study. Of the 32 patients who did not have a Day 5 6MWT, 27 were due to hospital discharge at day 2 or 3, and only 5 were due to worsening clinical condition. No adverse events were reported during the performance of the 6MWT. Four adverse events were reported in 4 patients during the 4 h period after the completion of the 6MWT—1 episode of paroxysmal atrial fibrillation with onset ~12 min after the start of the 6MWT, 2 episodes of fever (16–10 min after 6MWT start), and one episode of acute-on-chronic respiratory failure, 104 min after the start of the 6MWT. No early withdrawal of patients occurred. 

### 3.3. 6MWT Performance

**Figure 2:**
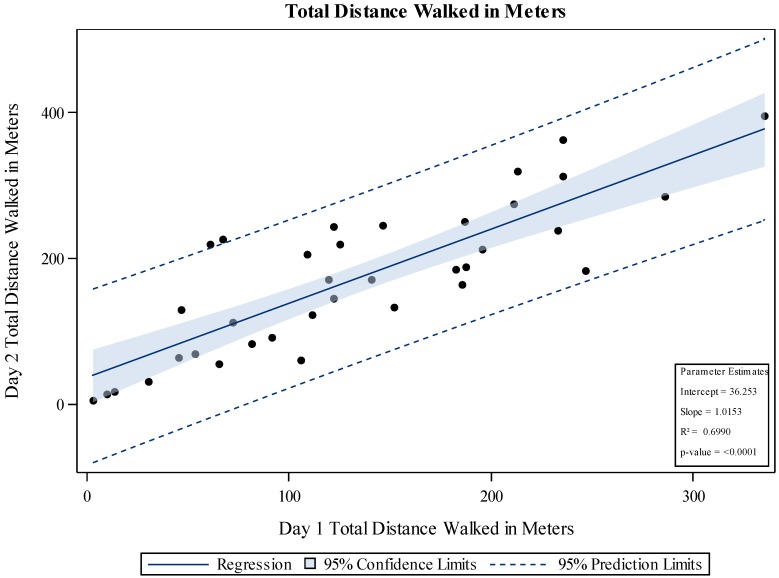
Mean six-minute walk test (6MWT) distance on Day 1 correlated with 6MWT distance on Day 2.

**Figure 3:**
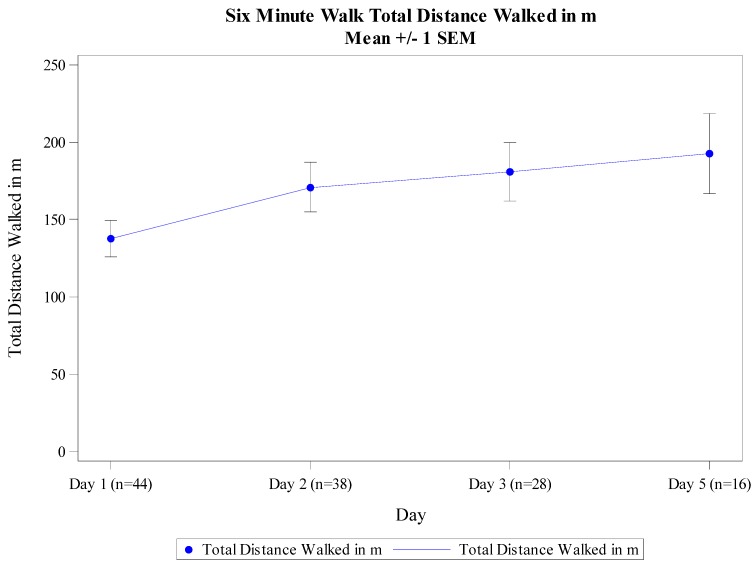
6MWT distance (meters) from day 1 through the end of Day 5. Only 16 subjects were still hospitalized at Day 5.

**Figure 4:**
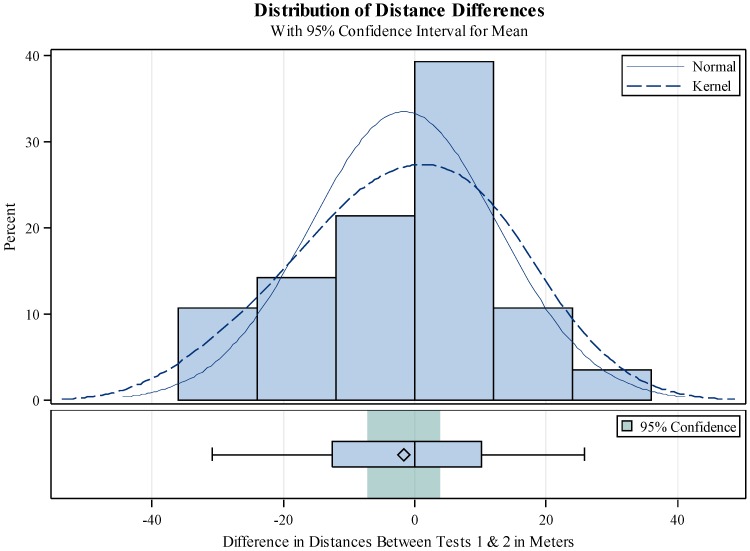
Difference (in meters) between duplicated 6-min walk tests on Day 3.

At baseline (Day 1), the patients walked a mean of 137.3 ± 78 m. There was no correlation between the Day 1 total distance walked or the distance to first stop with either LVEF or baseline SBP. On Day 2 (24 h after the first baseline 6MWT), the mean distance walked was 170.9 ± 100 m (Figure 2). On Day 3 (48 h after the first 6MWT) the mean distance walked was 180.8 ± 98 m and improved with each day in the hospital (Figure 3). The 6MWT demonstrated good reproducibility, as the distance walked by the 28 patients on the first 6MWT on Day 3 was similar to the distance on the repeated 6MWT on the same day (total distance walked 180.8 ± 98.3 m vs. 179.6 ± 96.2 m and distance to first stop 165.5 ± 110 m vs. 172.2 ± 102.2 m ) (Figure 4). Similar daily improvements in distance to the first stop were observed. Day 1 mean distance to the first stop was 112.0 ± 91 m, with an improvement to 152.5 ± 113 m (41.7 ± 71.7 m; *p* = 0.0013) and 164.4 ± 112 m (55.3 ± 83.8 m; *p* = 0.0025) on Days 2 and 3, respectively. 

### 3.4. 6MWT Correlation with Biomarkers and Outcomes

Day 1 BNP was not correlated with baseline 6MWT total distance (*r*^2^ = 0.044, *p* = 0.23) or distance to first stop (*r*^2^ = 0.047, *p* = 0.22). Changes in the 6MWT from day 1 to day 3 and changes in BNP over the same time did not correlate. Changes in the total distance and distance to first stop from Day 1 to Day 3 exhibited a non-significant trend toward an inverse correlation with length of stay of the index admission (*r* = −0.34, *p* = 0.09). Exploratory analyses for the relationship between the baseline 6MWT and the probability of readmission (*n* = 9 patients) within 30 days suggest that a higher baseline 6MWT was associated with a lower likelihood of readmission at 30 days (*p* = 0.06). 

## 4. Discussion

The results of our pilot study suggests the following: (1) the performance of a 6MWT is feasible for AHF patients, starting in the ED and continuing throughout hospitalization, as a minority of the 154 individual 6MWT’s were unable to be performed due to the worsening clinical status or patient refusal; (2) the 6MWT distance improves as patients receive AHF therapy during hospitalization; and, (3) the 6MWT distance is reproducible, as suggested by similar total distances when duplicate 6MWT testing occurred in the same subject within the same day. Six minute walk test performance in these patients may be a function of the patient’s baseline status, a response to therapy, or both. When compared to other critically ill HF patients, these AHF patients appear to have a lower baseline 6MWT distance (mean 182 m vs. 137 m respectively), and a smaller variability (SD of 140 m vs. 78 m, respectively) [11]. At lower 6MWT distances, the primary limitation to ambulation may be the patient’s physical status, and ambulatory distance may be less susceptible to verbal encouragement.

These pilot results suggest that the 6MWT could be utilized as a patient-centric endpoint in AHF studies. Our study suggests that the 6MWT is feasible in AHF patients, as early as 2.1 h after presentation (mean 10.8 h). Even patients who may be unable to walk at the baseline may improve sufficiently over their hospitalization, enabling the subsequent 6MWT assessments to be feasible. Thus, it would be important to include all of patients who indicate that they can walk at baseline, even if when they are approached for enrollment they are too acutely ill to perform a 6MWT. Similarly, when patient’s 6MWT time worsens or they become unable to perform the test, this could be a sign of a lack of response to therapy. It is currently unclear if an absolute minimum distance or a relative improvement over baseline 6MWT distance might be an appropriate predictor of other clinical outcomes. Larger studies are needed to determine whether the 6MWT distance is associated with both short- and long-term outcomes. While we saw a trend toward an association between 6MWT distance and 30-day events, we only had 9 patients who experienced 30-day readmission. As patients received AHF therapy, their walk distance improved, suggesting that this could be an objective marker to determine the optimal endpoints of therapy. If the 6MWT is found to be feasible in a larger cohort of patients, and associated with clinical events, collaboration across hospitals, and between investigators would be important to ensure its successful implementation.

Compared to the RED ROSE studies, where 1/3rd of patients either were unable or unwilling to perform bedside stepping in place, the 6MWT was indeed feasible in our cohort of AHF patients [12]. While the reasons to perform an initial 6MWT are multifactorial, the baseline differences between our study and RED ROSE are considerable. Our patients’ mean age was over 10 years younger, with a much higher proportion of African Americans, and a 10 mmHg higher mean systolic blood pressure. However, enrolling subjects in the ED setting supports the feasibility of performing functional testing acutely. Our pilot study, albeit limited by the small number of patients, also highlights the heterogeneity of the AHF population. 

While we were limited by the sample size, we did not see an association between changes in natriuretic peptide levels and 6MWT distance. There could be several reasons for this. Natriuretic peptide levels have wide daily variation and are a lagging indicator when considering AHF resolution. In stable outpatients with chronic HF, significant increases in BNP are not necessarily predictive of AHF. Further, tailoring therapy based on natriuretic peptide levels have yielded mixed results. Similarly, even significant increases in BNP levels in stable outpatients with HFrEF have not been predictive of subsequent AHF [13]. There was significant variability in natriuretic peptide levels within each patient in our study, and a decrease was not observed in all patients despite treatment and symptom improvement. This has been seen in prior ED-based AHF studies where serial BNP levels were measured. There was a mean change of 200–350 pg/mL in BNP during hospitalization, yet none of these changes were helpful in predicting hospital LOS, 30-day readmission, or all-cause mortality [14].

Limitations: While our pilot study suggests that the 6MWT is feasible and reproducible in AHF patients there were several limitations to consider. We enrolled a relatively small number of patients who experienced few 30-day events. We also only enrolled HFrEF patients as this study was designed to use the 6MWT in a subsequent clinical trial of HFrEF patients. There may be differences in HFrEF and HFpEF patients related to the changes in their 6MWT as they receive AHF therapy. Our clinical sites had a large proportion of younger, African American patients, so incorporating a more demographically diverse population of patients to ensure generalizability would be important. If illness severity or comorbidities differ in future studies than this may impact the proportion of patients who could complete all of the administered tests. Our study’s small sample size limited our ability to investigate this. The number of people who did a test on any given day is not the same as the number of people who did all 3 days of tests, as different patients missed different days. We did not track the reasons for the refusal to be in the study, and it would be good to quantify the impact of disease severity on patient participation in future studies.

In summary, our pilot study suggests that the 6MWT is both feasible and safe in patients with AHF. The majority of patients were able to perform this test, starting in the ED, and continuing throughout hospitalization. A larger study is necessary to evaluate its utility in a more demographically diverse population of patients, including those with preserved ejection fraction, and determine its relationship to longer-term outcomes such as HF readmission and mortality. 

## Figures and Tables

**Table 1:** Demographics and baseline characteristics of the study cohort.

Parameter	*N* * (%)	Mean (SD)
*Age*	48	55.6 (13.0)
*Gender*		
Male	32 (67)	
Female	16 (33)	
*Race*		
White	13 (27)	
Black/African-American	35 (73)	
*Primary HF Etiology*		
Ischemic	15 (31)	
Dilated	18 (28)	
Non-ischemic	11 (23)	
Chemotherapy	2 (4.2)	
Idiopathic	1 (2.1)	
Combined Ischemic(MI) & Non-ischemic(HTN)	1 (2.1)	
*NYHA Class*		
III	43 (90)	
IV	3 (6.3)	
Unknown	2 (4.2)	
*History of Angina*		
Yes	8 (17)	
No	40 (83)	
*LVEF by echocardiogram*	48	24 (9.1)
*eGFR*	46	71 (22.0)
*Baseline SBP (mmHg)*	48	127 (19)
*Baseline DBP (mmHg)*	48	78 (15)
*Baseline HR (bpm)*	48	86 (16)
*Weight (kg)*	47	92.8 (30.1)
IV Diuretics	41 (65)	
Topical/Sublingual Nitroglycerin	7 (15)	

* Two patients who left AMA within 24 h of presentation were included in this Table but excluded from subsequent analyses for having only one data point.
